# Unmasking of Partial Diabetes Insipidus during Stress but Not Maintenance Dosing of Glucocorticoids in an Infant with Septo-Optic Dysplasia

**DOI:** 10.1155/2011/817954

**Published:** 2011-03-16

**Authors:** Mala Puri, Anita Azam, KarenJ Loechner

**Affiliations:** 1 Division of Pediatric Endocrine, Department of Pediatrics, University of North Carolina, Chapel Hill, NC 27599, USA

## Abstract

*Background*. It is well acknowledged that glucocorticoid (GC) replacement can unmask diabetes insipidus (DI) in subjects with hypopituitarism. *Objective*. To increase the awareness and monitoring for transient and symptomatic DI in children with partial hypopituitarism during periods in which increased GC needs are required. *Methods/Case*. A 2-month-old female infant with septo-optic dysplasia (SOD; on thyroid and maintenance GC replacement therapy at 8 mg/m^2^/day) developed transient DI during 2 separate episodes of stress (one hypothermia, one febrile) when stress dosing of GC (25 mg/m^2^/day) was instituted. *Conclusion*. Children not diagnosed with DI during initial evaluation for hypopituitarism may benefit from rescreening of serum sodium levels during acute periods of stress that demand "stress" GC dosing. This will permit treatment and/or increased vigilance for ensuing permanent DI.

## 1. Background

Septo-optic dysplasia (SOD) is a malformation syndrome in which at least 50% of children have associated hypopituitarism [1, 2]. This condition includes agenesis of the septum pellucidum, hypoplasia, or aplasia of the optic nerves and chiasm that results in various degrees of visual impairment and abnormality of the hypothalamus causing secondary hypopituitarism [3]. Diabetes insipidus (DI) is a condition characterized by excretion of large volumes of dilute urine secondary to either a deficiency in the production/release of the hormone arginine vasopressin (AVP) that is synthesized in the hypothalamus and transported and stored in the posterior pituitary for release in response to a rise in plasma concentrations of osmotically active substances or an impaired response or resistance to AVP at the level of the kidney (nephrogenic DI, 4). The most common form of DI is due to a primary deficiency of AVP. Central DI due to midline brain abnormalities may also be accompanied by a defective thirst mechanism [4]. 

Here we present an infant in whom DI was not present during maintenance GC replacement but was unveiled during two episodes in which "triple stress GC dosing" was accompanied by temporary DI.

## 2. Methods/Case

Patient is a 2-month-old female with SOD diagnosed in the newborn period secondary to hypoglycemia and hypotonia. SOD was confirmed by both MRI and ophthalmologic exam. Hypopituitarism manifested with central hypothyroidism and hypoadrenalism such that her medications at presentation included thyroid supplementation (37.5 mcg or 10 mcg/kg/day) and hydrocortisone (8 mg/m^2^/day).

**Figure 1:**
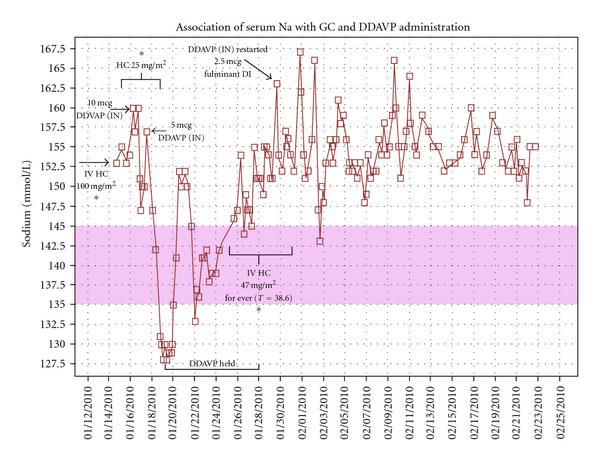
**Association of serum Na levels with (1) GC dosing and then (2) DDAVP administration over time.** The effect of stress HC dosing and DDAVP on serum Na levels in a patient with SOD. As shown on the  axis, serum Na (levels mmol/L, □) rise on each occasion in which HC dosing is increased over maintenance dosing (*), consistent with an unmasking of DI in child with partial hypopituitarism over time ( axis). Note that during a prolonged afebrile period and maintenance dosing, serum Na levels remained in the normal range (pink bar). In this patient, the transient DI during febrile episodes and GC stress dosing was then followed by progression to permanent DI with serum Na of 163 mmol/L, U_SG_ of 1.005, and an AVP level of <0.6 pg/mL, and patient was then maintained on DDAVP. Please note that the DDAVP doses were intranasal (IN) preparations given orally, so the effective DDAVP dose is 1/10 of the listed dose.

On day of life no. 70, she presented to the ED at the OSH with apnea and lethargy (which responded to stimulation), dehydration, and hypothermia (temperature 33.78). She underwent a septic workup, including urine and blood cultures, that were negative. Of note, her initial serum sodium (Na) was 153 mmol/L (range 135–145 mmol/L). She received a dose of ceftriaxone and an initial stress dose GC of 25 mg (100 mg/m^2^) at the OSH and then transferred to our PICU. Repeat serum Na was then 155 mmol/L, and she was treated with a normal saline intravenous (IV) fluid bolus and then maintained on D5.5 NS at maintenance for a 12-hour period. Her Na rose to 160, and she was given DDAVP (10 mcg of intranasal solution given orally; effective dose of 1 mcg). During this time she continued GC stress dosing of 2.3 mg hydrocortisone every 8 hours (at 25 mg/m^2^/day; see Figure 1).

Approximately 10 hours later, while on oral feeds and no supplemental IVF, her Na level dropped to 147 (rate of 1.2 mmol/hour). Oral feeds continued without DDAVP and, after 17 hours, her Na level again rose to 157 mmol/L. A second lower dose of DDAVP (effective dose of only 0.5 mcg) was then given as she appeared dehydrated and was tachycardic. Accurate urine output was not obtained as patient had not been catheterized. Surprisingly, this second dose resulted in an acute drop in her Na to 130 mmol/L over twelve hours (2.25 mmol/hour) and was accompanied by seizure activity. The patient was on oral feeds by this time, with IVF at 2 mL/hr to maintain IV patency. Her serum sodium levels normalized to 136–142 mmol/L over the following 18-hour period with fluid restriction.

Once normothermic, she was returned to her maintenance GC dose, and, of note, no subsequent doses of DDAVP were required during this 6-day period. At this time, a modified water deprivation test was performed and, at time of 15 hours, the patient's serum Na remained normal at 141 with a U_SG_ of 1.014, arguing against fulminant DI.

A subsequent febrile episode (temp 38.6°C) led to reinstitution of stress GC dosing (at 47 mg/m^2^/day) with repeat blood and urine cultures being negative. Again, her serum Na increased to a peak of 163 mmol/L and was accompanied by a U_SG_ of 1.005, a weight loss of 1.72 kg as well as an AVP level that was notably undetectable at <0.6 pg/mL (Mayo Clinic, Rochester, MN), supporting the diagnosis of another episode of "unmasking" of DI. Consequently, DDAVP was re-instituted, albeit at a markedly conservative dose of 2.50 mcg of IN preparations given orally (effective dose of 0.25 mcg) that successfully maintained her Na levels within a range of 145–155 mmol/L. Interestingly, she has been maintained on this dose since that time, given that her "transient" episodes seen during the increased GC exposure ultimately progressed to permanent, although likely "partial," DI.

## 3. Results/Conclusions

Although the "unmasking of DI" upon institution of GC replacement in children with pituitary insufficiency is well recognized, the mechanism of the sequelae is less well delineated. Both a GC-induced decrease in AVP release as well as inhibition of action at the level of the kidney has both been reported [5]. For example, Yamada et al. [6] demonstrated that corticotropin releasing hormone (CRH) administration augmented plasma arginine vasopressin (AVP) in response to an osmotic stimulus. The authors also reported that cortisol administration to patients with hypopituitarism decreased plasma AVP response to the osmotic stimulus [6]. Consequently, the authors deduced that the effect of cortisol was due to a central suppression of the AVP response to an osmotic stimulus. Furthermore, given that basal plasma renin activity, plasma aldosterone, plasma osmolality, hematocrit, body weight, mean blood pressure, and heart rate were similar in subjects with and without cortisol administration, the authors proposed that the effect of cortisol may be related to suppression of AVP release in response to osmotic stimuli. Furthermore, when the authors then pretreated the patients with DI with 20 mg of hydrocortisone, they found no significant change in the basal AVP levels; however, the maximal AVP response to an osmotic stimulus was significantly attenuated, thereby suggesting that CRH suppression by GCs was via inhibition of the AVP release [6]. In contrast, Linas et al. [7] measured total urine volume and U_SG_ in adrenalectomized rats and found that GC deficiency impairs renal water excretion. Moreover, Linas and colleagues demonstrated that GC-treated rats had a significantly higher urine flow (GFR) [7]. 

To date, no cases have been reported in which DI was induced only when greater than maintenance dosing of GCs were given. In our patient, DI was unmasked initially during a presumed viral illness only during episodes where stress dose GCs were required, suggesting a mild/partial DI, especially given the markedly low dose of DDAVP required to control her DI despite unmeasurable endogenous AVP levels during period of elevated serum Na levels and low U_SG_. Interestingly, after the continued need for stress GC dosing, permanent, although likely still partial, DI evolved with low dosing requirement, suggesting that we may have unmasked the progression of natural development to DI that was occurring but was brought to medical attention during her periods of increased GC requirements. 

This case supports, therefore, that, in children who have not been diagnosed with DI during their initial evaluation for hypopituitarism (e.g., SOD), rescreening of serum Na levels (along with parallel U_SG_ or osm along with body weight monitoring) during episodes of acute stress and concomitant increased GC dosing may, indeed, be clinically relevant by unveiling a partial/complete DI. Such vigilance could then obviate symptomatic DI, particularly in a young infant or in a child whose thirst mechanism may be either difficult to assess (since feeds are still formula) or may be impaired. It is also important to note, since DI could still be partial as it was in our patient, that the dosing of DDAVP is done conservatively and only when the patient has clinical signs of DI (e.g., hypernatremia accompanied by dehydration and/or weight loss). Finally, the DI should also be viewed as transient/episodic until permanent DI has been declared.

## Abbreviations

AVP:

Arginine vasopressin

DDAVP:

Desmopressin acetate

DI:

Diabetes insipidus

ED:

Emergency Department

GC:

Glucocorticoid

HC:

Hydrocortisone

osm:

Osmolarity

OSH:

Outside Hospital

PICU:

Pediatric Intensive Care Unit

SOD:

Septo-optic dysplasia

U_SG_:

Urine specific gravity.
